# *Paenibacillus larvae* bacteriophages: obscure past, promising future

**DOI:** 10.1099/mgen.0.000329

**Published:** 2020-01-28

**Authors:** Philippos K. Tsourkas

**Affiliations:** ^1^​ School of Life Sciences, University of Nevada Las Vegas, Las Vegas, NV, USA

**Keywords:** *Paenibacillus larvae*, American foulbrood, honeybee, bacteriophage, genomics, Amidase

## Abstract

*
Paenibacillus larvae* is a Gram-positive, spore-forming bacterium that is the causative agent of American foulbrood (AFB), the most devastating bacterial disease of the honeybee. *P. larvae* is antibiotic resistant, complicating treatment efforts. Bacteriophages that target *P. larvae* are rapidly emerging as a promising treatment. The first *P. larvae* phages were isolated in the 1950s, but as *P. larvae* was not antibiotic resistant at the time, interest in them remained scant. Interest in *P. larvae* phages has grown rapidly since the first *P. larvae* phage genome was sequenced in 2013. Since then, the number of sequenced *P. larvae* phage genomes has reached 48 and is set to grow further. All sequenced *P. larvae* phages encode a conserved *N*-acetylmuramoyl-l-alanine amidase that is responsible for cleaving the peptidoglycan cell wall of *P. larvae *. All *P. larvae* phages also encode either an integrase, excisionase or Cro/CI, indicating that they are temperate. In the last few years, several studies have been published on using *P. larvae* phages and the *P. larvae* phage amidase as treatments for AFB. Studies were conducted on infected larvae *in vitro* and also on hives in the field. The phages have a prophylactic effect, preventing infection, and also a curative effect, helping resolve infection. *P. larvae* phages have a narrow range, lysing only *P. larvae *, and are unable to lyse even related *Paenibacillus* species. *P. larvae* phages thus appear to be safe to use and effective as treatment for AFB, and interest in them in the coming years will continue to grow.

**Impact Statement**

Honeybees (*Apis mellifera*) are the producers of honey and key pollinators of many commercial crops, such as cherries and almonds. The global population of honeybees is currently in decline, due to a combination of factors. Among these is the disease known as American foulbrood (AFB), caused by the bacterium *Paenibacillus larvae *. Despite its name, AFB is a global problem. AFB has long been treated with antibiotics, but antibiotic-resistant strains of *P. larvae* are now widespread. A promising novel treatment for AFB are bacteriophages (viruses that infect bacteria) that target and destroy *P. larvae *. Phages have several advantages over antibiotics, such as being harmless to honeybees and humans, and co-evolving with their host to overcome resistance. Phages that infect *P. larvae* have been known since the 1950s, but interest in them has grown rapidly since the rise of antibiotic-resistant strains of *P. larvae *. This review presents the state of the art in *P. larvae* phage biology and efforts to treat AFB using phages. Early results show that *P. larvae* phages are a promising treatment for AFB, and the next few years should see increased interest in these phages and their use to treat AFB.

## Introduction

The disease known as American foulbrood (AFB), caused by the spore-forming, Gram-positive bacterium *Paenibacillus larvae,* is the most destructive bacterial disease of the common honeybee, *Apis mellifera* [1]. *P. larvae* spores are highly infectious to honeybee larvae (adults are immune), with ten spores sufficient to trigger a lethal infection [1, 2]. Infection typically occurs when food contaminated with spores is fed to a larva by worker bees. The spores germinate in the larval gut roughly 12 h after ingestion and proliferate rapidly, killing the infected larva [1, 3]. The infected larva decomposes into a brown liquid known as a ‘ropy mass’, which hardens to form scales containing millions of infectious spores [1, 4]. The spores are spread around the hive by worker bees removing the deceased larva, triggering a massive infection that eventually results in the annihilation of the hive’s larvae and the collapse of the hive. *P. larvae* spores are extremely durable, remaining infectious for decades, and are easily spread by the wind and during material exchanges between hives [1, 5]. AFB outbreaks were traditionally controlled using antibiotics such as oxytetracycline and tylosin tartrate; however, antibiotic-resistant *P. larvae* strains are now widespread [6–9]. Given its lethality and contagiousness, AFB is a notifiable disease. Currently the only method for combating outbreaks is wholesale hive incineration, which places great financial burden on beekeepers [1]. *P. larvae* possesses the attributes of a deadly pathogen, as it destroys hive after hive. However, the current situation also presents opportunities for developing alternative preventatives and treatments for AFB. Prominent among these is the use phages that infect *P. larvae,* the topic of this review.

## *P. larvae* history and biology

A disease of honeybees described as ‘foulbrood’ has been recorded from the mid-18th century [1]. In 1906, the American microbiologist George F. White isolated a bacterium from deceased honeybee larvae that he identified as the causative agent of foulbrood disease, and named it *Bacillus larvae* [10]. In 1950, a bacterium dubbed *Bacillus pulvifaciens* (‘powder scale’) that caused symptoms similar to those caused by *B. larvae* was isolated from infected honeybee larvae [11]. With the advent of 16S rRNA sequencing technology, *B. larvae* and *B. pulvifaciens* were grouped together into the new genus *Paenibacillus* as *Paenibacillus larvae* and *Paenibacillus pulvifaciens* [12]. In 1996, these were renamed as *P. larvae* subspecies *larvae* and *P. larvae* subspecies *pulvifaciens* [13]. In 2006, however, using enterobacterial repetitive intergenic consensus (ERIC) primer technology, it was shown that the two subspecies were in fact very similar to each other and were grouped into a single species, *P. larvae* [14]. Thus, it must be taken into account when searching the literature that *P. larvae* will appear under different names depending on the time period [1]. The current state of the art in *P. larvae* biology is that there are four *P. larvae* genotypes, with the ERIC I and ERIC II genotypes corresponding to the former *B. larvae,* and ERIC III and ERIC IV corresponding to the former *B. pulvifaciens* [1]. ERIC III and ERIC IV have not been isolated in recent years and are found only in archival stocks, and ERIC II is restricted to Europe [1]. ERIC II shows higher lethality than the other genotypes (7 days compared to 12 days to kill larvae), although the slower course of action of ERIC I makes it the more destructive of the two at the hive level [1]. As a result, the vast majority of AFB outbreaks, especially in the USA, are caused by the ERIC I genotype.

## *P. larvae* phage history

The first *P. larvae* phages were isolated in the 1950s, by N. Smirnova at the Leningrad Veterinary Institute, Russia (from dead larvae) [15], and by T.A. Gochnauer at the Canada Department of Agriculture (from lysogenic cultures) [16]. Several more isolates were characterized in North America and Eastern Europe in the following decades, mostly from lysogens [17–25]. All of these studies were characterization studies, and no phage genomes were sequenced at the time, as genome sequencing was either unavailable or prohibitively expensive. Moreover, at the time, AFB was routinely treated with antibiotics and, thus, interest in *P. larvae* phages remained scant. However, this began to change with the emergence of antibiotic-resistant strains of *P. larvae* in the early 2000s.

The first *P. larvae* phage to have its genome sequenced was phiIBB_Pl23, isolated in Portugal in 2013 [26], followed by phage HB10c2 in Germany in 2015 [27], and phages Diva, Lily, Rani, Redbud, Shelly, Sitara and Tripp, isolated in North Carolina [28, 29]. At the same time, 30 *P *. *larvae* phages were isolated at the University of Nevada, Las Vegas (UNLV), USA; the genome sequences of 9 of these were published in 2015 [30]. Concurrently, a large number of *P. larvae* phages were isolated over the period 2014–2106 by students at Brigham Young University (BYU) in Utah, USA, as part of BYU’s Phage Hunters course, and the genomes of 26 of these were published in 2018 [31, 32]. Included in this group is phage PBL1c, isolated from a lysogen by Dingman *et al*. in 1984 [22], and sent to BYU for sequencing in 2016 [31]. An additional four phage genomes from UNLV were published in 2018 [33], bringing the number of published *P. larvae* phage genomes to 48. The first comparative analysis of *P. larvae* phage genomes was published in 2016 [34], followed by a comprehensive genomic analysis of the 48 sequenced phage genomes in 2018 [35].

## Isolation sources

*P. larvae* phages have been isolated from sources such as hive interior, soil underneath healthy hives, infected larvae, lysogens and even commercial beeswax products [15–33]. The phages were isolated using standard phage isolation methods. The most detailed isolation protocols can be found in papers published by Beims *et al*. and Yost *et al*. [27, 36].

## Life cycle

All known *P. larvae* phages are temperate; to date no purely lytic *P. larvae* phages have been discovered. In addition, there are no known purely lysogenic *P. larvae* phages: all isolated *P. larvae* phages are able to lyse their host *in vitro*. However, there is wide variation in the lytic ability of the various *P. larvae* phages. Some phages, such as Halcyone, Fern and Willow, are strongly lytic of all *P. larvae* strains, while others, such as Xenia, are strongly lytic of one strain, but are unable to lyse others [36]. Burst size is typically of the order of~125 phage particles per infected host cell [27]. *P. larvae* phages have a narrow host range; they are unable to lyse bacteria closely related to *P. larvae *, such as *Paenibacillus alvei* and *Paenibacillus polymyxa* [36], although phage HB10c2 did show limited lytic activity against some other members of the genus *Paenibacillus* [27]. A narrow host range is an important consideration for using phages as treatment for AFB, as it indicates that *P. larvae* phages are unlikely to harm important honeybee gut flora.

## Morphology

**Fig. 1.**
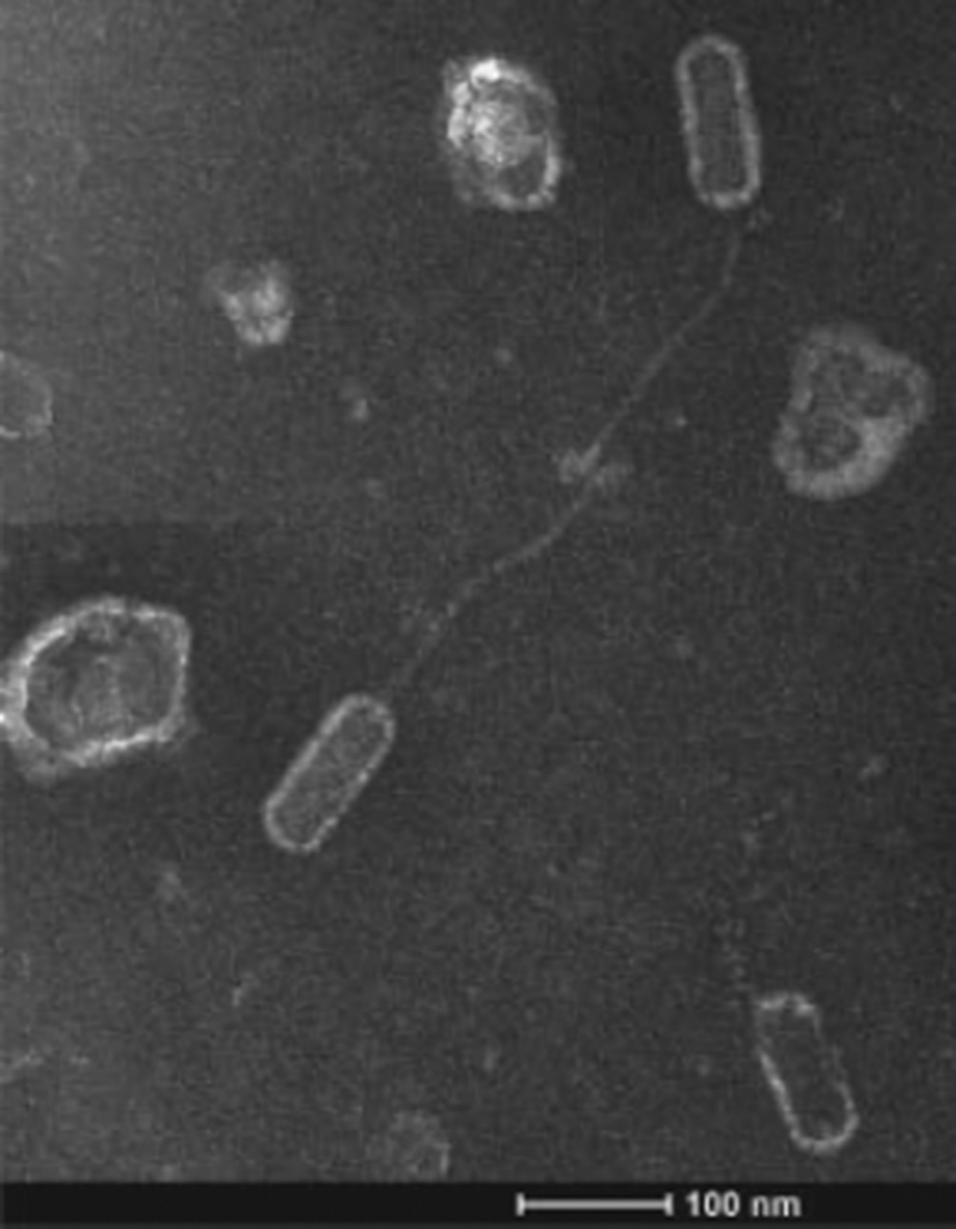
Transmission electron micrograph of a *P. larvae* phage, showing the *Siphoviridae* morphology characteristic of *P. larvae* phages, with a prolate capsid of 100×50 nm and a filamentous non-contractile tail of ∼150 nm in length, Reproduced from Yost *et al*. [36].

Some of the earliest *P. larvae* phage micrographs are from Dingman *et al*. [22]. Additional micrographs can be found elsewhere [27, 34–36]. The vast majority of *P. larvae* phages that have been imaged are of the *Siphovirus* morphotype, with flexible, non-contractile tails (Fig. 1). Tail length is in the 150–200 nm range [27, 34–36]. Tail length is proportional to the length of the tail tape measure gene, which varies between 2514 and 3705 bp [35]. Most *P. larvae* phages that have been imaged have prolate capsids of approximately 100×50 nm [21, 27, 34–36], but some phages have icosahedral capsids roughly 80 nm in diameter [35]. A major capsid protein was identified in all sequenced *P. larvae* phage genomes. The major capsid protein is distributed into four family clusters based on amino acid sequence identity [35]. However, it does not appear that this distribution correlates with capsid morphotype; there are instances of phages with different capsid morphologies having similar major capsid proteins, and phages with divergent major capsid protein sequences having similar capsid morphologies [35]. No other capsid proteins have been identified.

## Systematics

**Fig. 2.**
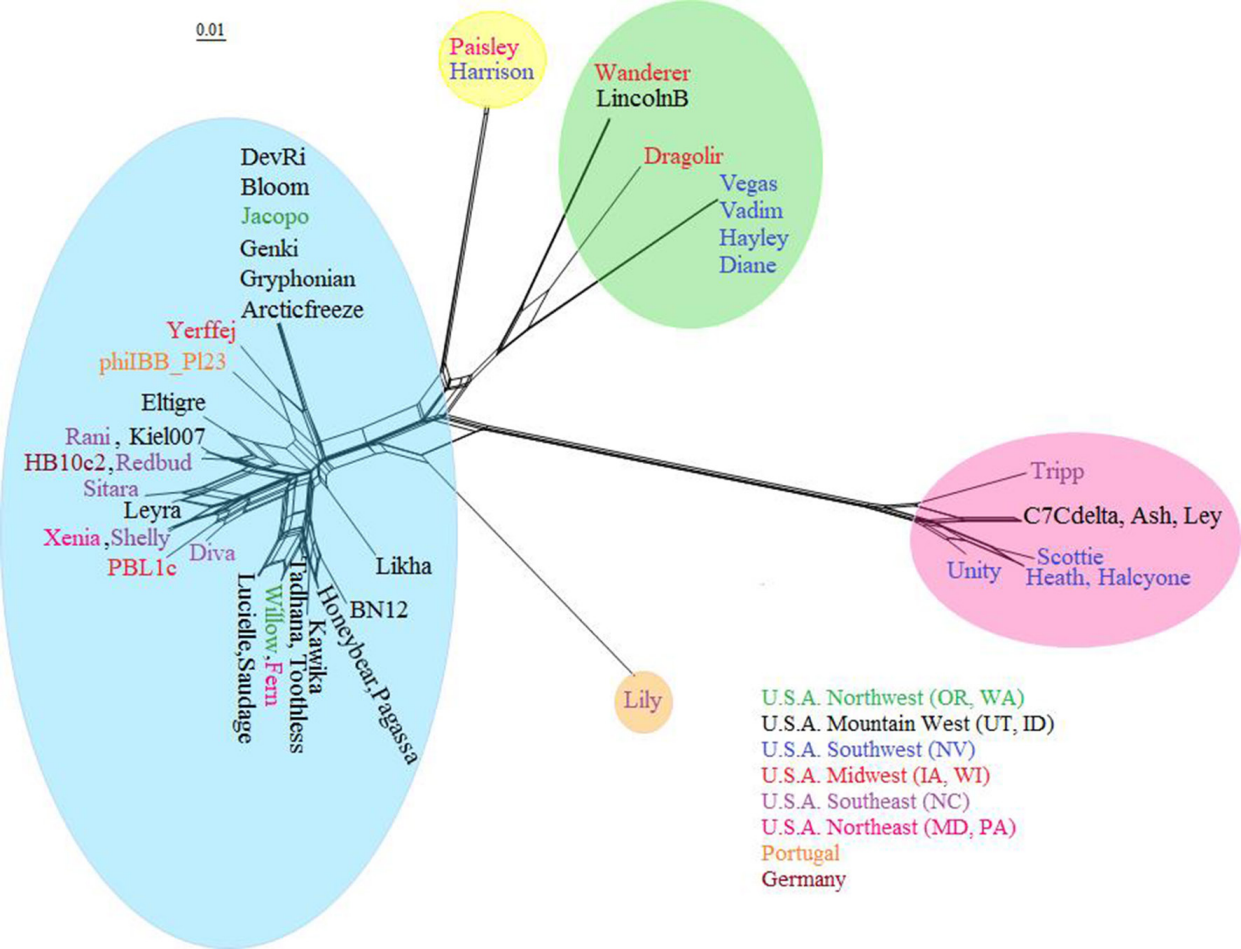
Phylogeny of all 48 sequenced *P. larvae* phages based on shared gene content, obtained with Splitstree [51]. Clusters are highlighted by colour. The Fern cluster (blue) is the largest; the Vegas cluster (green) is the most heterogeneous; the Halcyone cluster (pink) is the most distant; the Harrison cluster (yellow) contains only two phages; phage Lily (peach) is a singleton. Phage names are coloured by geographical origin. All clusters contain phages from different locations. The scale bar is inversely proportional to percent shared gene content.

*P. larvae* phages have been grouped into similarity clusters based on whole-genome nucleotide sequence identity as determined by ClustalW, using a cut-off value of 60 % for clusters and 90 % for subclusters [34, 35]. The clusters obtained with this method match those delineated using shared gene content, dotplots and pairwise genome maps [35]. A phylogenetic tree of the 48 sequenced *P. larvae* phages based on shared gene content is shown in Fig. 2 [35]. The phages are distributed into four similarity clusters and one singleton. Rather than an alphanumeric naming scheme, clusters were named after a representative phage [35]. The largest is the Fern cluster, which contains 30 of the 48 sequenced *P. larvae* phages, and in turn contains four subclusters and five in-cluster singletons [35]. All phages in this cluster have more than 60 % nucleotide sequence identity with each other [35]. The Harrison cluster consists of only two phages, Harrison and Paisley, which differ by only one gene [35]. The Vegas cluster is the most heterogeneous, consisting of two dissimilar subclusters (less than 50 % identity) that are joined together by phage Dragolir, which has more than 60 % nucleotide sequence identity with members of both subclusters [35]. Phage Lily is a singleton, with less than 50 % nucleotide sequence identity to any of the other phages [35]. The Halcyone cluster is the most distant from all the others, with less than 30 % nucleotide sequence identity to any other cluster [35]. It is not known whether the large disparity in cluster size reflects the underlying reality of *P. larvae* phage biology, or whether it is due to sampling bias. Two taxonomy proposals have been ratified by the International Committee for Taxonomy of Viruses, and that number is certain to grow in the future [37, 38].

Of note is that geographical origin is not correlated with genome sequence similarity [35], which can be seen in Fig. 2. There are instances of phages from widely different locations having very high nucleotide sequence identity (e.g. phages Xenia and Shelly, phages Fern and Willow, phages Diane and Vegas, and phages Redbud and Kiel007 all have more than 99.5 % nucleotide sequence identity with each other) [35]. Even phages isolated on different continents are found in the same subcluster (e.g. HB10c2 and Kiel007, Rani or Redbud) [35]. However, phages from the same location and even the same isolation source can be very dissimilar from each other (e.g. phages Diva, Lily and Tripp from North Carolina, and phages Halcyone and Harrison from the Las Vegas area) [35]. It is currently not known why geography has no correlation with genomic sequence, especially considering that the ERIC I strain of *P. larvae* is much more common in the Western hemisphere, and the ERIC II strain is restricted to the Eastern hemisphere [1].

## Genome characteristics

A summary of the genome properties of *P. larvae* phages is shown in Table 1, with more detailed information in the paper by Stamereilers *et al*. [35]. Phages in the Halcyone cluster have genomes in the 50–56 kbp range, while all other phages have genomes in the 35–46 kbp range. The Halcyone cluster phages have higher G+C content, in the 48–49 mol% range, while all other phages are in the 40–44 mol% range. The number of genes scales linearly with genome length, with the Vegas subcluster phages are the most gene dense and have the highest coding fraction.

**Table 1.** Summary of genome characteristics of each of the five *P *. *larvae* phage clusters The quantities in each column represent means (except for the number of phages in each cluster). DNA packaging strategies are cohesive ends (cos), either 3′ or 5′, and direct terminal repeats (DTR).

Cluster	No. of phages	Genome length (kbp)	G+C content (mol%)	No. of genes	No. of genes per kbp	Coding fraction (%)	DNA packaging mechanism
**Fern**	30	39 291	41.8	66	1.68	90.9	3’ cos
**Harrison**	2	44 209	40.1	78	1.76	91.5	3’ cos
**Vegas**	7	43 319	43.3	78	1.79	93.8	3’ cos
**Lily**	1	44 952	42.7	75	1.67	90	5’ cos
**Halcyone**	8	55 071	48.4	88	1.59	91.2	DTR

A total of 3462 genes were identified in the 48 *P *. *larvae* genomes, with a mean of 72 genes per genome [35]. The longest gene is 3705 bp, and the shortest 75 bp, with a median gene length of 375 bp [35]. Nine genes longer than 3000 bp were identified, and 21 shorter than 100 bp, with 20 % of genes in the 200–300 bp range [35]. The genomes were annotated using the method described in detail by Salisbury *et al*. [39].

**Fig. 3.**
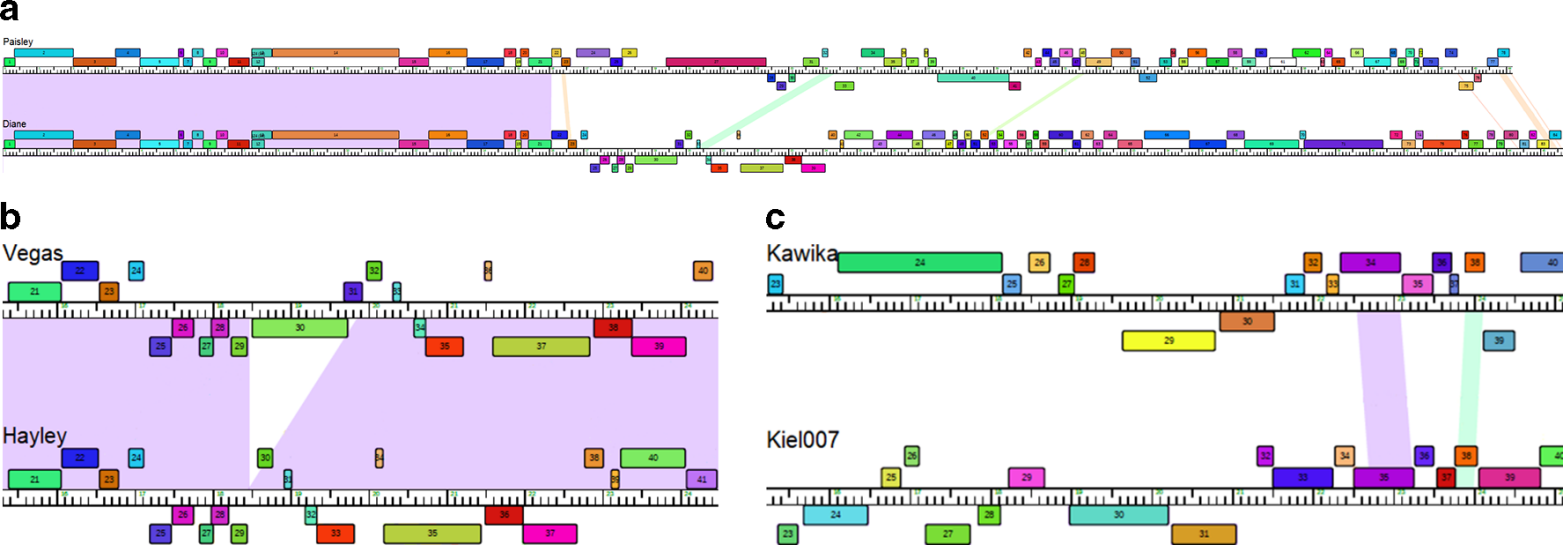
Pairwise phage genome maps made with Phamerator [52]. Genes are shown as coloured boxes; homologous genes are shown in the same colour; genes are shown above or below the ruler depending on which strand they are located; conserved regions between the genomes are shaded in various colours. (a) The front section of genomes of phages Paisley and Diane is conserved, while the middle and rear sections are not. (b) An instance of gene insertion/deletion; genes 30 and 31 in the genome of phage Vegas are absent in the genome of phage Hayley, in a region where the two genomes are conserved. (c) Two genes conserved between the genomes of phages Kawika and Kiel007 (gene 34/35 and gene 38) are located in an otherwise non-conserved region between the genomes.

Pairwise genome maps show that the front section of the genomes is conserved between phages in the same cluster, and sometimes even between phages belonging to different clusters, while the middle and rear sections of the genomes are divergent [34, 35]. Fig. 3(a) shows phages Diane and Paisley, which belong to different clustershowever, the front third of their genomes is conserved. Pairwise genome maps also reveal cases of phages that are missing genome sections that are present in other phages in the same cluster [34, 35]. Fig. 3(b) shows genes 30 and 31 in phage Vegas are missing in phage Hayley, indicating a likely deletion event. Pairwise genome maps also show instances of isolated, conserved genes in regions that are not conserved between phages in the same cluster, possibly indicating horizontal gene transfer [34, 35]. Fig. 3(c) shows genes 34 and 38 in phage Kawika are present in phage Kiel007 in a region where no other genes are conserved between the two phages, which are in the same cluster.

## Functional genomics

**Fig. 4.**
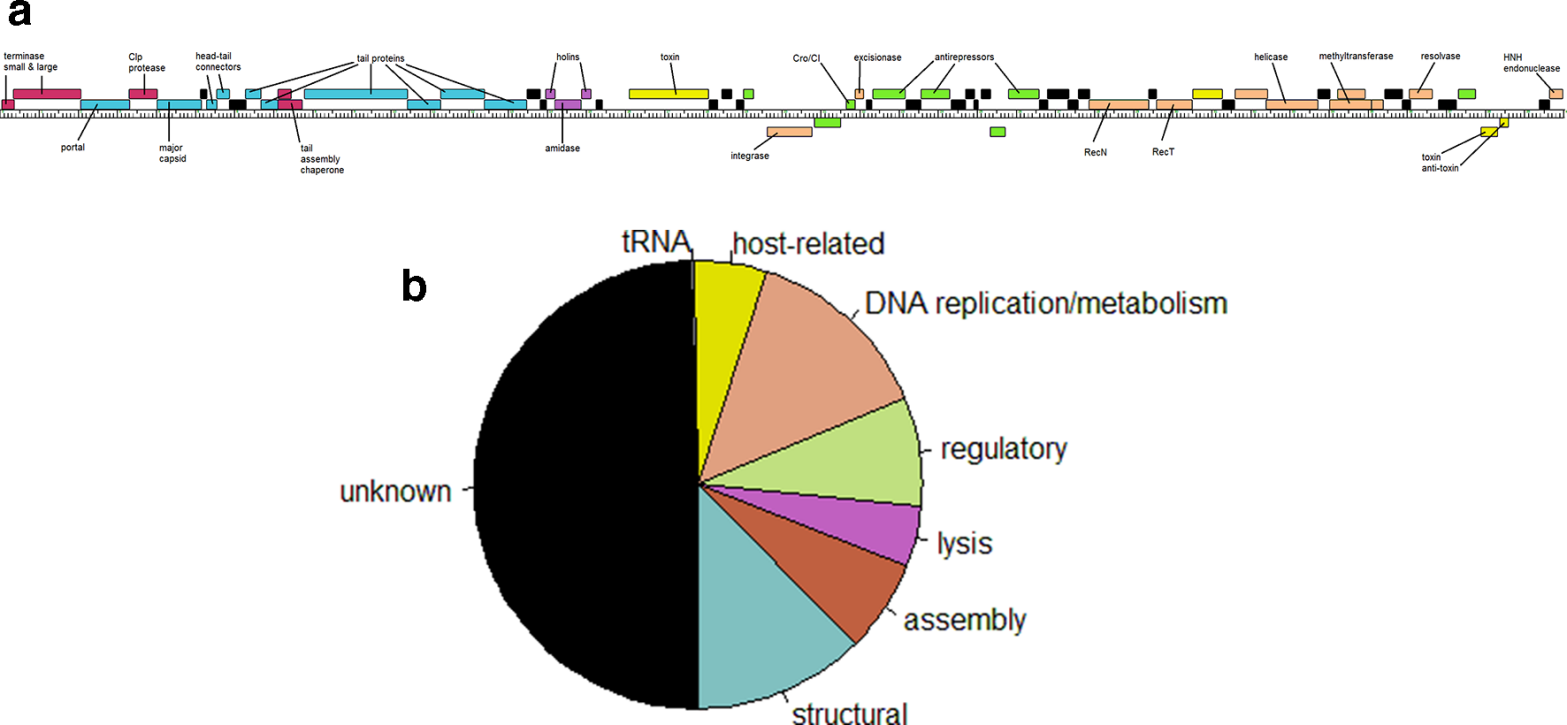
(a) Genome map of phage Pagassa, a typical Fern cluster phage, in which genes are coloured according to function (burgundy, assembly; blue, structural; purple, lysis; green, regulatory; peach, replication/DNA metabolism; yellow, host-related). (b) Distribution of putative functional categories of the genes in the genomes of six representative phages (Fern, Fern cluster; Harrison, Harrison cluster; Diane, Vegas cluster; Dragolir, Vegas cluster; Lily, Lily cluster; Halcyone, Halcyone cluster). About half the genes have putative functions; only one tRNA gene was found.

Approximately 90 % of *P. larvae* phage genes have sequence similarity matches to proteins in the National Center for Biotechnology Information non-redundant database [35]. Approximately 55 % of *P. larvae* phage gene products have at least one sequence similarity match to proteins with putative function [35]. Gene products with putative function were classified into seven categories: (1) structural; (2) virion assembly; (3) lysis; (4) regulatory; (5) DNA replication/metabolism; (6) host-related functions; and (7) tRNA [35]. Structural and assembly genes are located in the front of the genome and tend to be conserved within clusters; regulatory, DNA replication/metabolism and host-related genes are located in the mid and rear section of the genome and are not conserved even within clusters. Fig. 4(a) shows the genome of phage Pagassa, a typical Fern cluster phage, where the genes are colour coded by function type. The fraction each functional category comprises out of the all the gene products in the genomes of six selected representative phages is shown in Fig. 4(b).

Gene products with the following functions were identified in all *P. larvae* phage genomes: (1) small and large terminase; (2) portal protein; (3) Clp protease, (4) major capsid protein; (5) two tail assembly chaperone proteins; (6) tail tape measure protein; (7) *N*-acetylmuramoyl-l-alanine amidase; (8) two putative holins; and (9) several tail proteins [35]. As is common with most phages, virion particle and virion assembly genes are found in the front of the genome and tend to be conserved within clusters [35]. DNA replication/metabolism, regulatory and host-related genes are in the mid and rear section of the genome and tend not to be conserved, even within clusters [35]. A lone tRNA gene was found in the genome of phage Dragolir [35].

The large terminase is of particular interest because it packages phage DNA into empty capsids, and is used to infer a phage’s DNA packaging strategy [40]. The packaging strategy may be inferred by similarity to large terminases of phages whose DNA packaging strategy is known from experiments [40, 41]. Using this approach, the Halcyone cluster phages use the direct terminal repeats (DTR) strategy, while all other phages use the ‘cohesive ends’ (cos) strategy [35].

An integrase, excisionase, antirepressor or Cro/CI was found in all sequenced phage genomes, congruent with the observation that all sequenced *P. larvae* phages are temperate [35]. An integrase was found in all phage genomes except the Halcyone cluster, but these phages all contain a Cro/CI gene, indicating they are temperate as well [35]. Other commonly found functions include a major tail protein, head-tail connectors, bacterial toxins, a toxin–antitoxin system, various anti-repressors, a helicase, a resolvase, and an ArpU transcriptional regulator [35].

## Lytic mechanisms

**Fig. 5.**
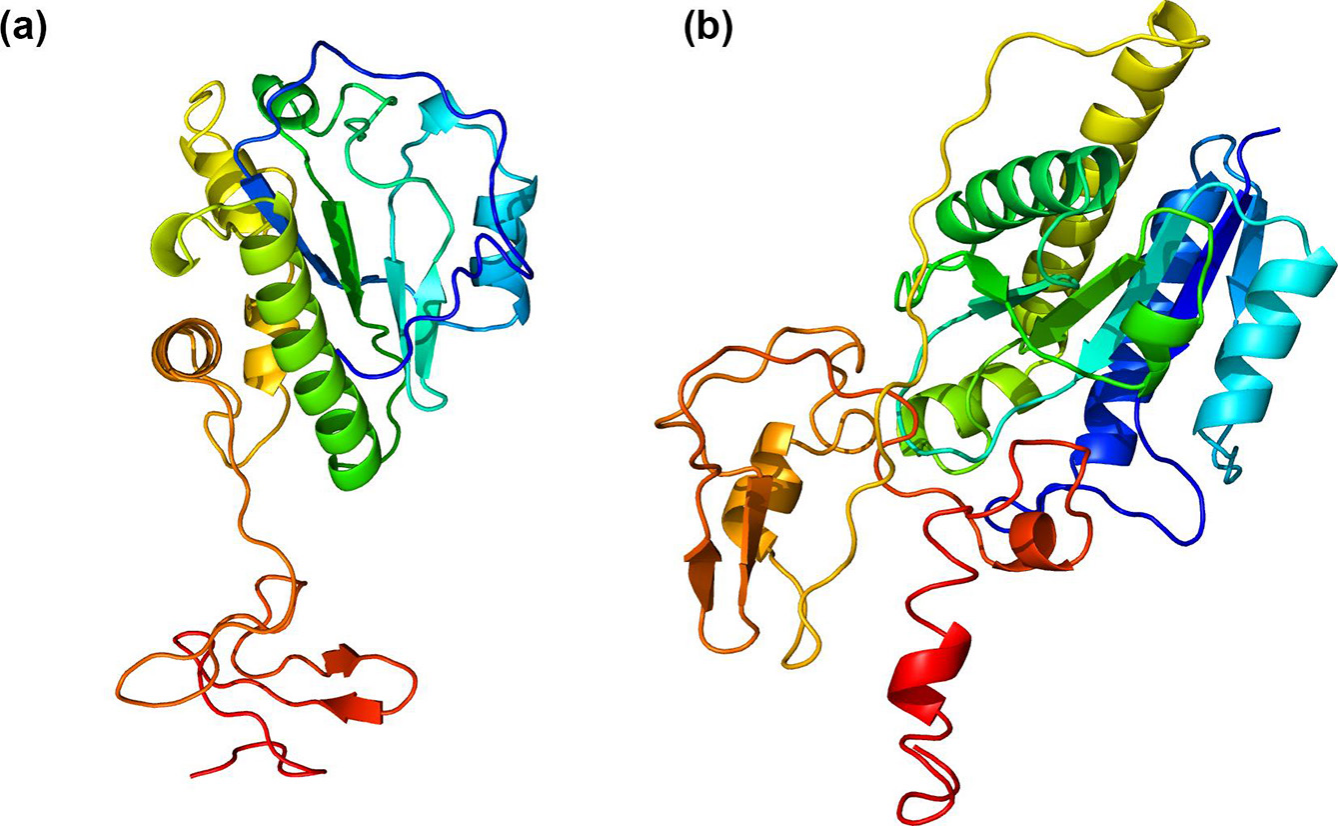
Predicted protein structure of the *N*-acetylmuramoyl-l-alanine amidase of phage Xenia (a) and phage Halcyone (b). The structures were obtained with Phyre2 using the ‘Intensive’ setting. The protein structures are coloured from the C-terminus (red) to the N-terminus (blue). The distinct C-terminal cell-wall binding domain and N-terminal catalytic domain are clearly distinguishable along with the linker connecting them. The amidase of phage Halcyone has a more complex structure.

Of great interest are the mechanisms by which *P. larvae* phages lyse their host. Tailed phages lyse their host by means of a holin/lysin cassette consisting of a hydrophobic holin protein that punctures the host’s inner plasma membrane and a hydrophilic lysin that cleaves the host peptidoglycan wall [42–44]. An *N*-acetylmuramoyl-l-alanine amidase has been identified in all sequenced *P. larvae* phage genomes [35]. It is the protein responsible for lysing *P. larvae,* by cleaving its peptidoglycan cell wall [45–47]. It is the most studied *P. larvae* phage protein, being the subject of at least three experimental studies, and the only *P. larvae* phage protein whose function has been experimentally verified [45–47]. The *N*-acetylmuramoyl-l-alanine amidase is distributed into two highly distinct similarity clusters, with amidases of the Halcyone cluster phages accounting for one cluster and the amidases of all remaining phages in the other cluster [35]. The two amidase clusters are very divergent, with no two amidases from different clusters having more than 13 % amino acid sequence identity with each other [35]. Within-cluster similarity is high, however, with all amidases having greater than 90 % amino acid sequence identity with all other amidases in their cluster [35]. The amidases of the Halcyone cluster phages are also 60 aa longer (~285 vs ~224 aa) [35]. The predicted protein structure obtained with Phyre2 [48] of the amidases of phages Xenia and Halcyone is shown in Fig. 5.

Given that phages from both clusters are competent at lysing *P. larvae *, it is currently not known why there exist two distinct amidase clusters, and the effect of these differences in terms of lysis. Despite the high degree of amino acid identity within clusters, there exist numerous point mutations within clusters [35]. These differences are significant, and likely explain why phages from the same cluster have different lytic profiles. For example, phage Fern strongly lyses all four *P. larvae* genotypes, but phage Xenia can only lyse the ERIC I genotype; their amidases differ by 10 residues (insertions or point mutations) [35, 36].

In a 2015 study, the amidase of phage phiIBB_Pl23 was shown to be highly effective at lysing the ERIC I and ERIC II genotypes of *P. larvae in vitro*, and also highly specific to *P. larvae *, failing to lyse other bacterial species [45]. The amidase was active across a range of biochemical conditions; most notably, it was more effective at lysis in high pH conditions [45]. However, the amidase was ineffective against dormant or germinating spores [45].

In a 2019 study investigating the amidase of phage PhiIBB_Pl23, it was shown that this protein consists of two domains: a N-terminal catalytic domain and a C-terminal cell binding domain (CBD) [46]. The CBD appears essential for lysis; truncated amidases consisting of only the catalytic domain were unable to lyse [46]. The CBD was shown to be highly specific to *P. larvae* and failed to bind to other bacterial species; thus, accounting for the specificity of *P. larvae* phage amidase to its host [46]. The CBD of PhiIBB_Pl23 showed weaker binding to the cell wall of ERIC III and ERIC IV genotypes compared to the ERIC I genotype, indicating the *P. larvae* genotypes have different cell walls [46]. The amidase of PhiIBB_Pl23 has high amino acid sequence identity (>90 %) to other *P. larvae* phage amidases, except those of the Halcyone cluster [35]; thus, the findings of this study are broadly applicable. Indeed, many *P. larvae* phages are better at lysing the ERIC I genotype than the ERIC III and ERIC IV genotypes [36].

**Fig. 6.**
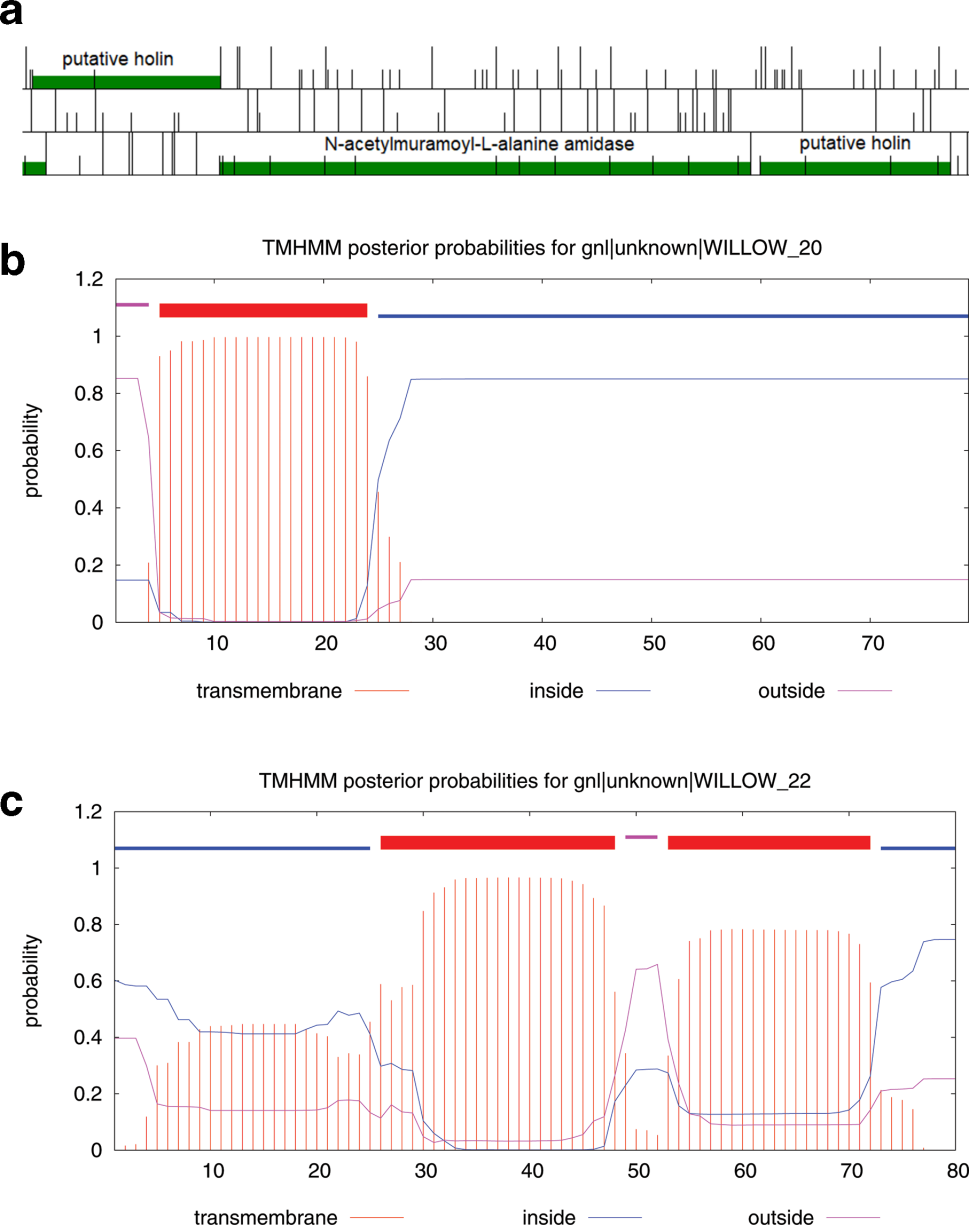
Location of the two putative holins and the *N*-acetylmuramoyl-l-alanine amidase in the genome of phage Willow, as visualized with the software DNA Master (http://cobamide2.bio.pitt.edu/computer.htm). (a) Coloured regions indicate ORFs; half-height vertical bars represent start codons; full height bars represent stop codons; each row corresponds to a reading frame. Start codons were chosen using the method described by Salisbury and Tsourkas [51]. The stop codon of the first putative holin overlaps with the start codon of the *N*-acetylmuramoyl-l-alanine amidase, while the second putative holin is in the same reading frame as the amidase; the three genes, thus, likely form an operon. (b, c) The graphical outputs from the transmembrane domain prediction program tmhmm (http://www.cbs.dtu.dk/services/TMHMM/) for the two putative holins are shown [53]. Red represents transmembrane domains, blue intracellular domains and purple extracellular domain. The first putative holin has one transmembrane domain (b), and the second putative holin two transmembrane domains (c).

Two putative holins have been identified in all *P. larvae* phage genomes [35]. One of these is immediately upstream of the amidase, and the other immediately downstream of the amidase (Fig. 6). Holins are generally not conserved and difficult to identify bioinformatically, and the sequence similarity matches are uncertain [35]; the first putative protein has similarity matches to ‘bacteriocin biosynthesis protein’ [35]. However, the fact that these genes form an operon with the amidase (Fig. 6a), and the fact that they also possess transmembrane domains (Fig. 6b, c), strongly suggests that they may indeed function as holins [35]. If both proteins have holin function, this raises the question as to why *P. larvae* phages have two holins. Alternatively, one of these may be an anti-holin or have some other function in regulating lysis.

## Treatment of AFB

Since 2015, several studies exploring the of use of *P. larvae* phages to treat AFB [27, 36, 49] in larvae reared *in vitro* and in hives [49] have been published. In two of these studies, honeybee larvae infected with *P. larvae* spores and subsequently treated with phages had significantly higher survival than infected larvae that were not treated with phages [36, 49]. In one study, three of the most strongly lytic phages in UNLV stocks (Fern, Willow, Xenia) were used individually [49]; in the other study, a cocktail of seven phages was used [36]. In both studies, the phages also had a prophylactic effect; larvae that were treated with phages and subsequently infected with *P. larvae* had significantly higher survival than infected control larvae that were not treated with phages [36, 49]. In one of the studies, prophylactic treatment resulted in significantly higher survival compared to post-infection treatment [49]. Importantly, both studies showed that the phages had no adverse effect on honeybee larvae or their gut microbiota: uninfected honeybee larvae exposed to phages had very similar survival rates to control larvae [36, 49]. The phages were highly specific to *P. larvae *, failing to lyse even closely related bacterial species such as *Paenibacillus lentimorbus *, *Paenibacillus popilliae *, *Paenibacillus polymyxa* and *Paenibacillus alvei* [36]. In another study, however, phage HB20c2 did not improve the survival of infected larvae, despite the fact that it lysed *P. larvae in vitro* [27]. Possible reasons for this could be the phage entering lysogeny, or being unable to access or penetrate *P. larvae* in the honeybee gut [27]. This suggests that phage cocktails may be necessary to treat AFB.

The first use of phages to treat AFB in hives was published in 2017 [50]. A total of 39 phages were isolated, 3 of which were selected to be in a treatment cocktail based on their lytic profiles. In preliminary tests, the phages were shown to be safe for use; healthy hives to which the phage cocktail was applied did not have significantly higher mortality than control hives. However, healthy hives mock-treated with the antibiotic tylosin tartrate had significantly higher mortality than control hives. In subsequent trials, the phage cocktail was applied prophylactically to five uninfected hives, with another five hives in a mock-treated control group. After 2 weeks, four of the five hives in the control group were infected with AFB, while the five phage-treated hives remained healthy. After the phage cocktail was applied to the infected hives, all four made a full recovery within 6 weeks. The results of this study show that *P. larvae* phages have both a prophylactic and a curative effect on hives infected with AFB [50].

Given that *P. larvae* is a Gram-positive bacterium, another possibility is using the amidase alone as a treatment for AFB [46]. In a 2015 study, the amidase of phage Xenia was isolated, cloned and used to treat honeybee larvae infected with *P. larvae* [47]. After 8 days, infected larvae that were treated with the amidase showed 75 % survival, compared to 90 % survival in the control group and 20 % survival in the infected but not treated group [47]. The amidase was highly effective at lysing ERIC I *P. larvae* genotypes, but not ERIC III and ERIC IV, exactly as found with phage Xenia [47]. Importantly, the amidase was highly specific to *P. larvae *, showing only slight lysis of related strains such as *P. polymyxa* and *P. lentimorbus *, and no lytic activity against any other bacterial species [47]. Using the amidase alone instead of the phage has the advantage that it is much more difficult for *P. larvae* to escape the amidase as opposed to escaping phages. Bacteria can evade phages by acquiring CRISPR sequences or mutating their receptors, but escaping the amidase would require mutating their peptidoglycan, a much more difficult task.

## Conclusions

In recent years, there has been increasing interest in phages that infect *P. larvae *, the bacterium responsible for the devastating AFB disease in honeybees. *P. larvae* phages were first isolated in the 1950s, with the first sequenced genome appearing in 2013. Since then, the number of sequenced *P. larvae* phages has increased to 48 and, given the potential of the phages to treat AFB, that number is set to grow in the coming years.

*P. larvae* phages are genomically diverse, distributed into four clusters and a singleton by genomic similarity. One cluster is very large, containing the majority of *P. larvae* phages (Fern), one cluster contains only two phages (Harrison), one cluster is heterogeneous (Vegas), while one cluster is very distant from all the others (Halcyone). Genome size ranges between 35 and 56 kbp and G+C content between 40 and 49 mol%. The Halcyone cluster phages have significantly longer genomes, have higher G+C content and use a different DNA packaging strategy than the other *P. larvae* phages. The clusters are distinct, with little sequence similarity between them. The current systematics picture is quite different from what it was in 2016 [34], and will doubtless change again in the future. Geographical origin does not correlate with sequence similarity.

*P. larvae* phages lyse their host by means of a conserved *N*-acetylmuramoyl-l-alanine amidase that has been identified in all phages and whose function has been verified through experiments. The *N*-acetylmuramoyl-l-alanine amidases are distributed into two distinct clusters, with one cluster containing the Halcyone cluster amidases and the other cluster containing the remainder. Differences in amino acid sequence of the amidase likely account for the different lytic profiles of the various phages.

All known *P. larvae* phages are temperate, but *P. larvae* phages have been shown to be effective in combating AFB both *in vivo* and in hives, without any discernible harm to honeybees. In contrast to antibiotics, phages are also unlikely to pose a threat to honeybee and human health. The amidase of *P. larvae* phages was also shown to be effective at clearing AFB, without the need for phages. With alternative methods coming up short and AFB continuing to be a global problem, *P. larvae* phages have the potential to be one of the main methods for combating AFB in the future, and interest in them will doubtless continue to grow. Nevertheless, more work is needed to establish the effectiveness and safety of *P. larvae* phages and phage amidases in combating AFB in the field.

Additional future directions of interest are: (1) identification of the function of more *P. larvae* phage proteins, especially using experimental approaches; (2) precise identification of the mechanisms by which *P. larvae* phages lyse their hosts, including identification of *P. larvae* phage holins and any additional lysins; (3) the role of phage-encoded toxins in *P. larvae* antibiotic resistance and virulence; (4) the mechanism by which *P. larvae* phages penetrate their host; (5) the mechanisms by which *P. larvae* phages enter and exit lysogeny; (6) identifying uses of *P. larvae* phage proteins for biotechnology applications; and (7) understanding how *P. larvae* defend against infection by phages.
